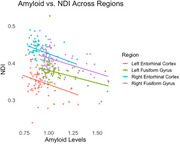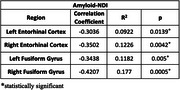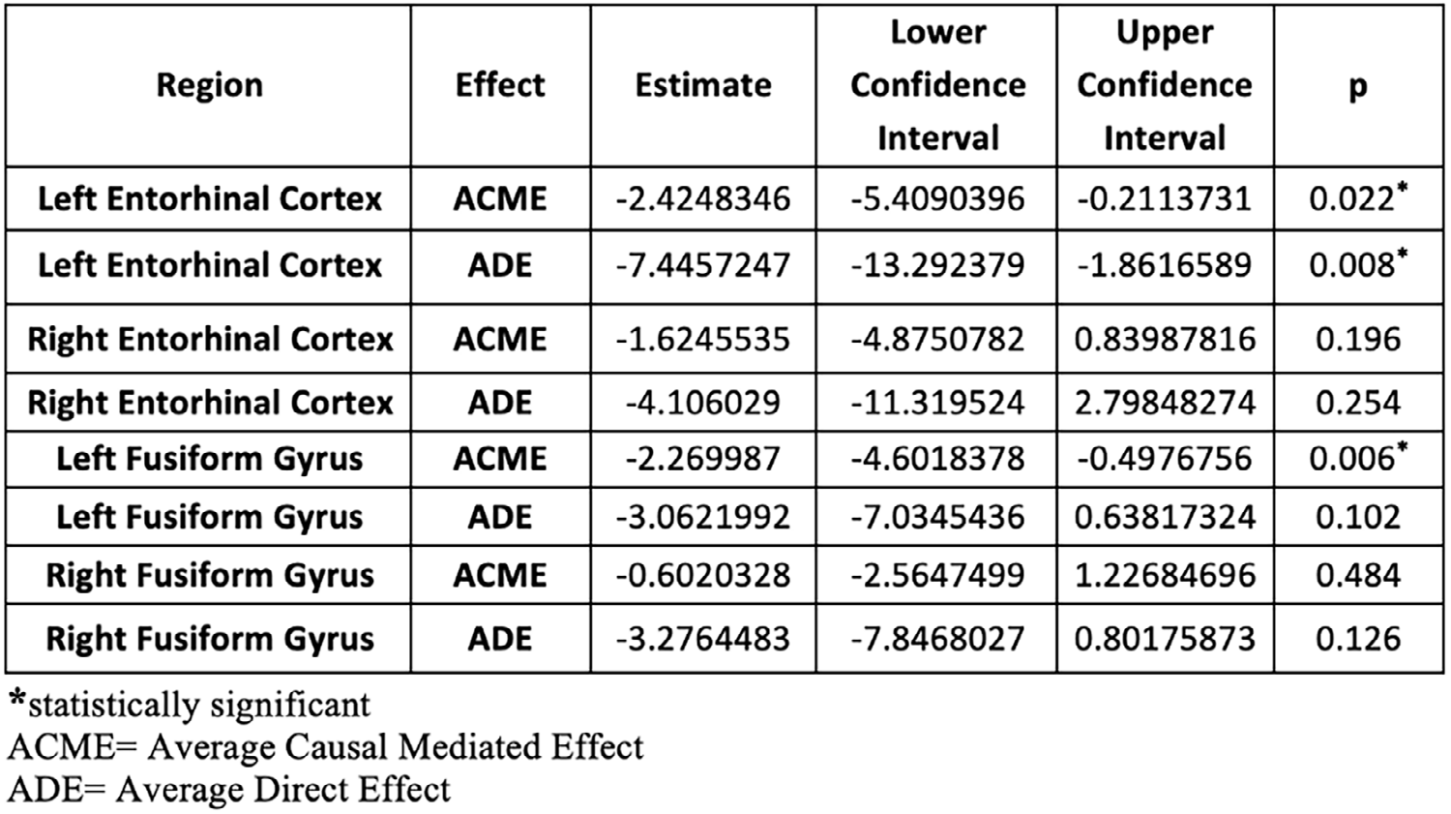# Neurite Degradation Mediates the Impact of Amyloid Deposition on Global Cognition in Unimpaired Older Adults

**DOI:** 10.1002/alz70856_103236

**Published:** 2025-12-26

**Authors:** Sasha Hakhu, Sydney Y Schaefer, Scott C Beeman

**Affiliations:** ^1^ Arizona State University, Tempe, AZ, USA

## Abstract

**Background:**

Prior studies suggest that amyloid deposition and microstructural brain changes, measurable via the diffusion magnetic resonance imaging (dMRI) metric neurite density index (NDI), are associated with Alzheimer's disease (AD). It is unclear, however, whether the relationship between amyloid and neurite density is present in asymptomatic older adults. This study therefore investigated the link between amyloid deposition and neurite degradation (reflected by lower NDI) in cognitively unimpaired individuals, and whether NDI mediates the relationship between amyloid levels and global cognition, particularly in the entorhinal cortex and fusiform gyrus, regions critical for memory and cognition.

**Method:**

We analyzed cognitively unimpaired older adults (*n* = 65; mean age 69.5±5.5, 45F) from the ADNI3 database. Advanced dMRI were processed to derive NDI and amyloid levels were obtained from PET imaging data. Regions of interest, including the entorhinal cortex and fusiform gyrus were selected a priori based on prior work. Here, correlation and mediation analyses assessed the relationship between amyloid, NDI, and global cognition (MoCA), with FDR correction and bootstrap simulations ensuring robust statistical evaluation.

**Result:**

Amyloid and NDI were negatively correlated in the entorhinal cortex (EC) and fusiform gyrus (FG). The left EC showed a correlation of ‐0.304 (R^2^ = 0.092, *p* =  0.014), and the right EC had a stronger correlation of ‐0.350 (R^2^ = 0.123, *p* =  0.004). The left FG exhibited ‐0.344 (R^2^ = 0.118, *p* =  0.005), and the right FG showed ‐0.421 (R^2^ = 0.177, *p* =  0.0005). Mediation analysis revealed significant effects for the left EC (Average Causal Mediated Effect, ACME, *p* =  0.02) and FG (ACME *p* =  0.006), but not for right regions (*p* > 0.05).

**Conclusion:**

This study showed amyloid deposition, particularly in the left entorhinal cortex and fusiform gyrus, is linked to microstructural degradation and cognitive decline. Significant negative correlations between amyloid and NDI suggest higher amyloid levels lead to greater neurodegeneration and lower cognitive scores even in asymptomatic individuals. Mediation analysis showed NDI partially mediates this relationship in the left entorhinal cortex and fusiform gyrus. These findings highlight early neurodegenerative changes in cognitively unimpaired individuals and emphasize the importance of amyloid and neurite density in understanding cognitive decline.